# An evaluation of the organ procurement and transplantation network's expanded post-transplant performance metrics

**DOI:** 10.3389/frtra.2023.1237112

**Published:** 2023-08-16

**Authors:** John S. Malamon, Bing Ho, Whitney E. Jackson, Jessica L. Saben, Jesse D. Schold, James J. Pomposelli, Elizabeth A. Pomfret, Bruce Kaplan

**Affiliations:** ^1^ Department of Surgery, University of Colorado Anschutz Medical Campus, Aurora, CO, United States; ^2^ Research and Education (CCTCARE), Colorado Center for Transplantation Care, Aurora, CO, United States; ^3^ Department of Medicine, University of Colorado Anschutz Medical Campus, Aurora, CO, United States; ^4^ Feinberg School of Medicine, Department of Medicine and Surgery, Northwestern University, Chicago, IL, United States

**Keywords:** transplantation, liver, lung, kidney, heart, performance metrics and evaluation

## Abstract

On July 14, 2022, the Organ Procurement and Transplantation Network's (OPTN) Membership and Professional Standards Committee (MPSC) approved bylaws including two new post-transplant performance evaluation metrics, the 90-day (90D) and 1-year conditional on the 90-day (1YC90D) graft survival hazard ratio (HR). These metrics have replaced the previous 1-year (1Y) unconditional, post-transplant graft survival HR and are used to nationally rank and identify programs for MPSC review. The MPSC's policies have major implications for all transplant programs, providers, and patients across the United States. Herein we show two significant limitations with the new evaluation criteria, arbitrary censoring periods and interdependence in the new performance metrics. We have demonstrated a strong and consistent inverse correlation between the new evaluation metrics, thus proving a lack of independence. Moreover, these two evaluation criteria are interdependent even at nominal HRs. Thus, the 90D cohort can be used to accurately predict whether the 1YC90D is above or below a given HR threshold. This could alter practice behaviors and the timing of patient event reporting, which may result in many unintended consequences related to clinical practice. Here we provide the first evidence that this new evaluation system will lead to a significant increase in the number of programs flagged for MPSC review. When this occurs, the cost of operating a transplant program will increase without a clear demonstration of an increased accuracy in identifying problematic programs.

## Introduction

If one were to flip a coin 365 times, you would very closely approximate a 50%–50% heads-to-tails ratio. Yet, within that apportionment there could easily be runs of five to six consecutive heads or tails. Thus, splitting the coin tosses into one run of 90-flips and one run of 275-flips would increase the chance of artifactual clustering. To separate these two processes, one would have to assume that the rate of events was truly different instead of randomly different between the first 90 coin tosses and the next 91 to 365 coin tosses (1). Recently, the Organ Procurement and Transplantation Network's (OPTN) Membership and Professional Standards Committee (MPSC) approved the inclusion of two new program-level, post-transplant performance evaluation metrics: the 90-day and 1-year conditional graft survival hazard ratio (2). The 90-day (90D) cohort is the graft survival evaluated from 1-day to 90-days post-transplant, while the 1-year conditional graft survival assumes that a patient survives the first 90 days and hence evaluates survival from 91-days to 1 year (1YC90D) post-transplant. More simply put, patient events occurring within the first 90 days of transplant are removed from the 1YC90D cohort. Given the example of the coin flips, there is a concern that by splitting up these two censoring periods one may create artifactual inferences between random events and systematic events. Systematic events are attributable to a program. To test this hypothesis, we analyzed the OPTN's national patient outcomes data to determine whether there was a “bright line” between the 90D and 1YC90D censoring periods and to assess the correlation between the two new performance metrics.

## Materials and methods

To test these hypotheses and measure the interdependence of the new performance metrics, we analyzed the OPTN's national post-transplant outcomes for all transplant centers using the Spring and Fall 2022 program-specific reports (PSRs). All data were downloaded from the OPTN's website, including all heart, kidney, liver, and lung post-transplant outcome metrics in the United States. We constructed two separate cohorts (Table 1) from these PSRs to perform this basic analysis. The Spring and Fall 2022 PSRs were the first to provide the 90D and 1YC90D patient outcomes.

**Table 1:** Summary of national transplant centers for the Spring and Fall 2022 program-specific reports.

		Spring 2022	Fall 2022
Recipient	Organ	Number of programs	Number of programs
Adult	All	1,119	1,115
Adult	Heart	128	125
Adult	Kidney	620	620
Adult	Liver	304	305
Adult	Lung	67	65
Pediatric	All	552	543
Pediatric	Heart	64	63
Pediatric	Kidney	317	313
Pediatric	Liver	150	147
Pediatric	Lung	21	20

The Spring and Fall 2022 cohorts consisted of 248 and 247 transplant centers, respectively.

The methods used herein are simplistic and intuitive. First, we calculated the baseline HRs for all adult and pediatric programs. The baseline HR was defined as the total number of observed (O) events plus 2 divided by the number of expected (E) events plus 2. OPTN uses a shrinkage factor of 2 to calculate the baseline HR (3). Supplementary Figures S1–6 provide the baseline HR distributions for all six cohorts. Next, we performed the one-sample Kolmogorov-Smirnov methodology (4) to test for normality in the log-transformed, baseline HR distributions. This is a well-accepted test for normality. We then constructed two-by-two contingency tables (Supplementary Figures S7 and S8) for programs with baseline HRs greater than five thresholds (1.0, 1.25, 1.5, 1.75, and 2.0) for the 90D and 1YC90D cohorts. These contingency tables were then used to calculate the correlation coefficients between the new metrics and the accuracy of using the 90D HR to predict the 1YC90D HR. Specifically, assuming a 90D HR threshold, we asked, can we accurately predict whether the 1YC90D HR will be above or below that same threshold? Accuracy was defined as the proportion of programs with HRs greater than the defined threshold among the total number of programs evaluated. In this way, we examined the interdependence of the new performance metrics as a function of the baseline HR.

## Results

We calculated the correlation coefficients between the two evaluation metrics by creating two-by-two contingency tables for programs with baseline HRs greater than five thresholds (1.0, 1.25, 1.5, 1.75, and 2.0) for the 90D and 1YC90D cohorts. The accuracy of using the 90D HR to predict the 1YC90D HR was used to determine the interdependence of the new evaluation metrics. The mean correlation coefficients between the 90D and 1YC90D cohorts showed a strong inverse correlation for all organs and ranged from −0.672 at HRs greater than 1.0 to −0.865 at HRs greater than 1.75 (Supplementary Figure S9). Next, the accuracy of using the 90D HR to predict the 1YC90D HR was also determined to demonstrate the interdependence of these metrics. The mean correlation coefficients showed a strong inverse correlation for all organs and ranged from −0.672 to −1.0. The prediction accuracy ranged from 0.63 to 0.94 (Table 2).

**Table 2:** Accuracy for predicting the 1-year conditional hazard ratio based on the 90-day hazard ratio at four thresholds for the Spring and Fall 2022 cohorts.

Hazard ratio threshold	Accuracy	Lower 95%	Upper 95%	*p*-value
Spring 2022				
1.0	0.630	0.607	0.654	0.001
1.25	0.718	0.695	0.739	0.001
1.5	0.876	0.859	0.892	0.001
1.75	0.941	0.928	0.952	0.001
Fall 2022				
1.0	0.628	0.604	0.651	0.001
1.25	0.711	0.688	0.733	0.001
1.5	0.867	0.849	0.883	0.001
1.75	0.936	0.923	0.947	0.006

The prediction accuracy of the 90D HR was highly consistent between the two cohorts and ranged from approximately 0.63 at hazard ratios above 1.0–0.94 at hazard ratios above 1.75.

In order to assess whether 90-days post-transplant was a “bright line” with an abrupt change in the rate of patient events, we assessed the difference between the HR of the 90D and 1YC90D cohorts. There was no statistically significant difference between the mean 90D (1.018) and 1Y (1.015) baseline HRs, suggesting that the 90-day censoring period is arbitrary. There was no difference in the mean 30-day HRs as compared to the 90D and 1Y baseline HRs. To emphasize the potential impact of the new metrics, we went back and found that the new performance metrics result in approximately twice the total number of programs with baseline HRs above 1.75 as compared to the previous standard. We did this by calculating the total number and percentage of programs with HRs above 1.75 for the two new metrics along with the previous standard (1Y). For the Spring 2022, we found that 70 or 4.19% (90D), 30 or 1.82% (1YC90D), and 52 or 3.11% (1Y) of programs had HRs above 1.75. For the Fall 2022, we found that 69 or 4.16% (90D), 40 or 2.44% (1Y90D), and 54 or 3.25% (1Y) of programs had HRs above 1.75.

## Discussion

These findings are important because they show that the selection of a 90D post-transplant censoring period is arbitrary and may lead to unintended consequences such as the false inference of systematic clustering vs. random events. We have demonstrated a strong and consistent inverse correlation between these two metrics, proving a lack of independence in these evaluation metrics. Critically, the 90D cohort can be used to accurately predict whether the 1YC90D is above or below a given HR threshold.

Due to artifactual clustering, empirical observation, and interdependence in the new evaluation metrics, we expect a significant increase in the number of programs identified for MPSC performance review. Because events are now parsed into smaller groups, there is an increased likelihood for a series of random patient events to occur within a given evaluation period. This also increases Type 1 error in identifying potentially problematic programs because random events are more likely to be associated with a program's performance. The second reason that we predict an increase in MPSC flagging is the result of the interdependence of the new evaluation metrics. Because they are inversely correlated, a program who was not likely to flag for the 90D criteria is now more likely to flag for the 1YC90D criteria, and *vice versa*.

Although we fully acknowledge the difficulty and nuance involved in successfully identifying problematic programs, the results and findings demonstrated herein illustrate the importance of ensuring that evaluation metrics are not interdependent, and the cutoff point is indeed a “bright-line”. Parsing post-transplant outcomes into two arbitrary groups mathematically increases a program's likelihood of being identified for MPSC review without clearly demonstrating an increase in accurately identifying problematic programs. For example, a program could have 100% patient survival for an entire year but be identified for review because of several random patient graft failures occurring in the 90D cohort that cannot be attributed to a program's practices or performance. Given the HHS and OPTN's mandate to increase the number of transplants performed nationally, we believe that the risk aversion engendered by these new performance metrics could be counterproductive to that end.

Based on these findings, we have two high-level recommendations for OPTN and MPSC. First, no two evaluation metrics should be dependent on each other. This interdependence increases error and allows programs who are close to flagging for one evaluation metric to easily predict the other metric. We are concerned that this will change programs' practice behaviors and therefore patient event reporting. Our second recommendation is to not use a 90D evaluation metric. In accordance with the null hypothesis, we do not support the implementation of the 90D evaluation criteria without additional evidence to show that it is consistently and well correlated with a program's performance over time. To more accurately identify problematic programs, we will need to better describe the relationship between the new metrics and programs' performance over extended time periods. Therefore, in our assessment, a 1Y evaluation periods alone is preferable. In conclusion, given the HHS and OPTN's mandate to increase the number of transplants performed nationally, we believe that the loss/risk aversion engendered by these new performance metrics could be counterproductive to that end. Our findings also suggest that these effects will not be equally felt across programs. Finally, these policies may also significantly drive up administrative and healthcare costs for programs and patients, which would adversely affect the entire transplant community.

## Data Availability

The original contributions presented in the study are included in the article/**Supplementary Material**, further inquiries can be directed to the corresponding author.
