# Supplementary material for: An evaluation of the organ procurement and transplantation network's expanded post-transplant performance metrics
Source: Front Transplant. 2023 Aug 16;2:1237112. doi: 10.3389/frtra.2023.1237112 (PMC11235232; doi:10.3389/frtra.2023.1237112)

Supplementary Appendix

Supplement to: An evaluation of the Organ Procurement and Transplantation Network's expanded post-transplant performance metrics

This appendix has been provided by the authors to give readers additional information about the work.

Supplementary appendix for:

An evaluation of the Organ Procurement and Transplantation Network's expanded post-transplant performance metrics

Contents

Supplemental Figure 1. 1-year Baseline Hazard Ratio Density, Spring 2022 3

Supplemental Figure 2. 90-day Baseline Hazard Ratio Density, Spring 2022 4

Supplemental Figure 3. 1-year Conditional Baseline Hazard Ratio Density, Spring 2022 5

Supplemental Figure 4. 1-year Baseline Hazard Ratio Density, Fall 2022 6

Supplemental Figure 5. 90-day Baseline Hazard Ratio Density, Fall 2022 7

Supplemental Figure 6. 1-year Conditional Baseline Hazard Ratio Density, Fall 2022 8

Supplemental Figure 7. Contingency Tables for 90-day versus 1-year Conditional Cohorts by Hazard Ratio, Spring 2022 9

Supplemental Figure 8. Contingency Tables for 90-day versus 1-year Conditional Cohorts by Hazard Ratio, Fall 2022 10

Supplemental Figure 9. Interdependence Between the New Post-Transplant Evaluation Metrics, Spring and Fall 2022 11

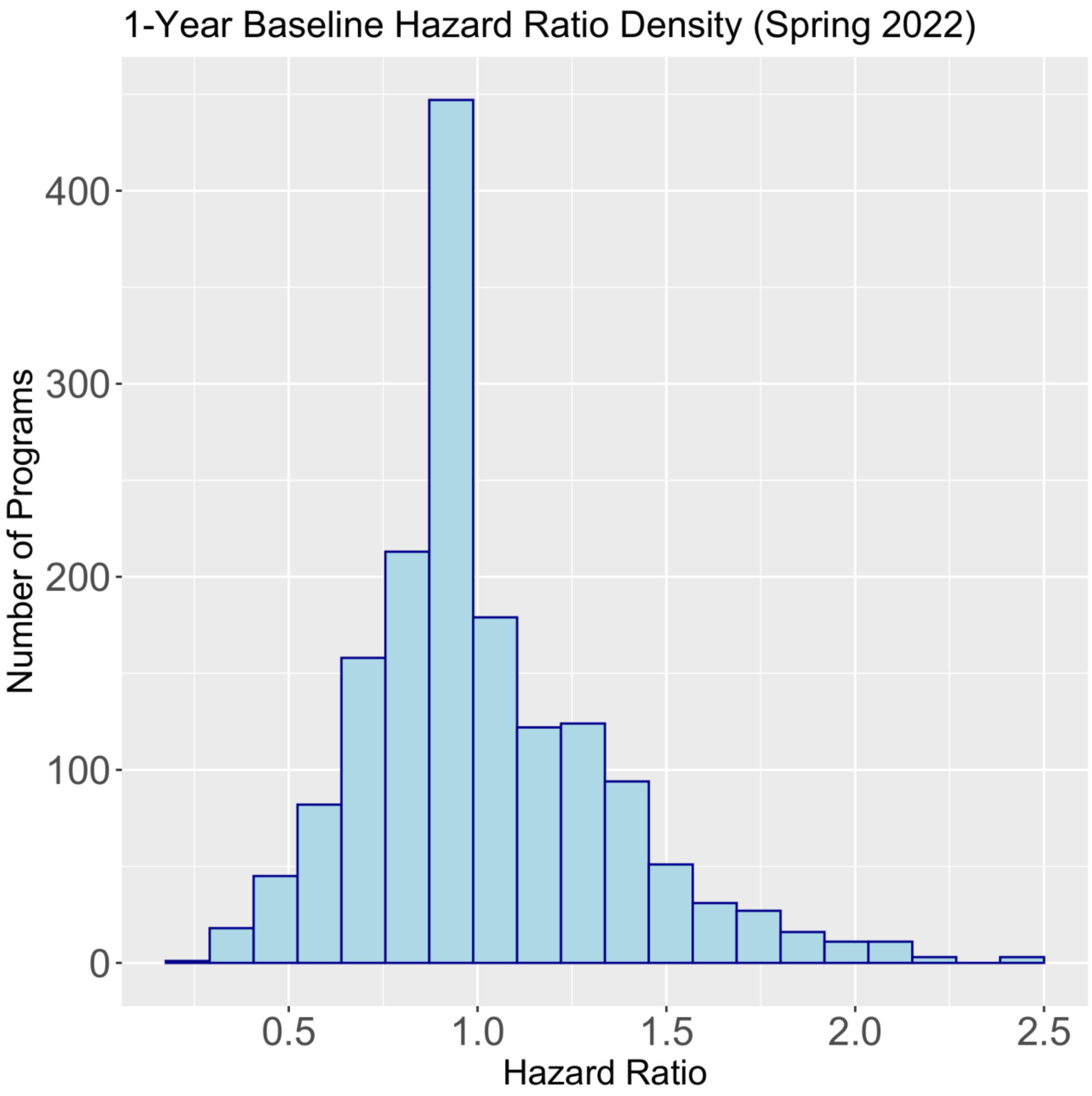

Supplemental Figure 1. 1-year Baseline Hazard Ratio Density, Spring 2022. Hazard ratios (HR) are provided for all programs (N=1,671). 1-year (1Y) hazard ratios ranged from 0.283 to 2.492 with a mean of 1.015, median of 0.959, and standard deviation of 0.324.

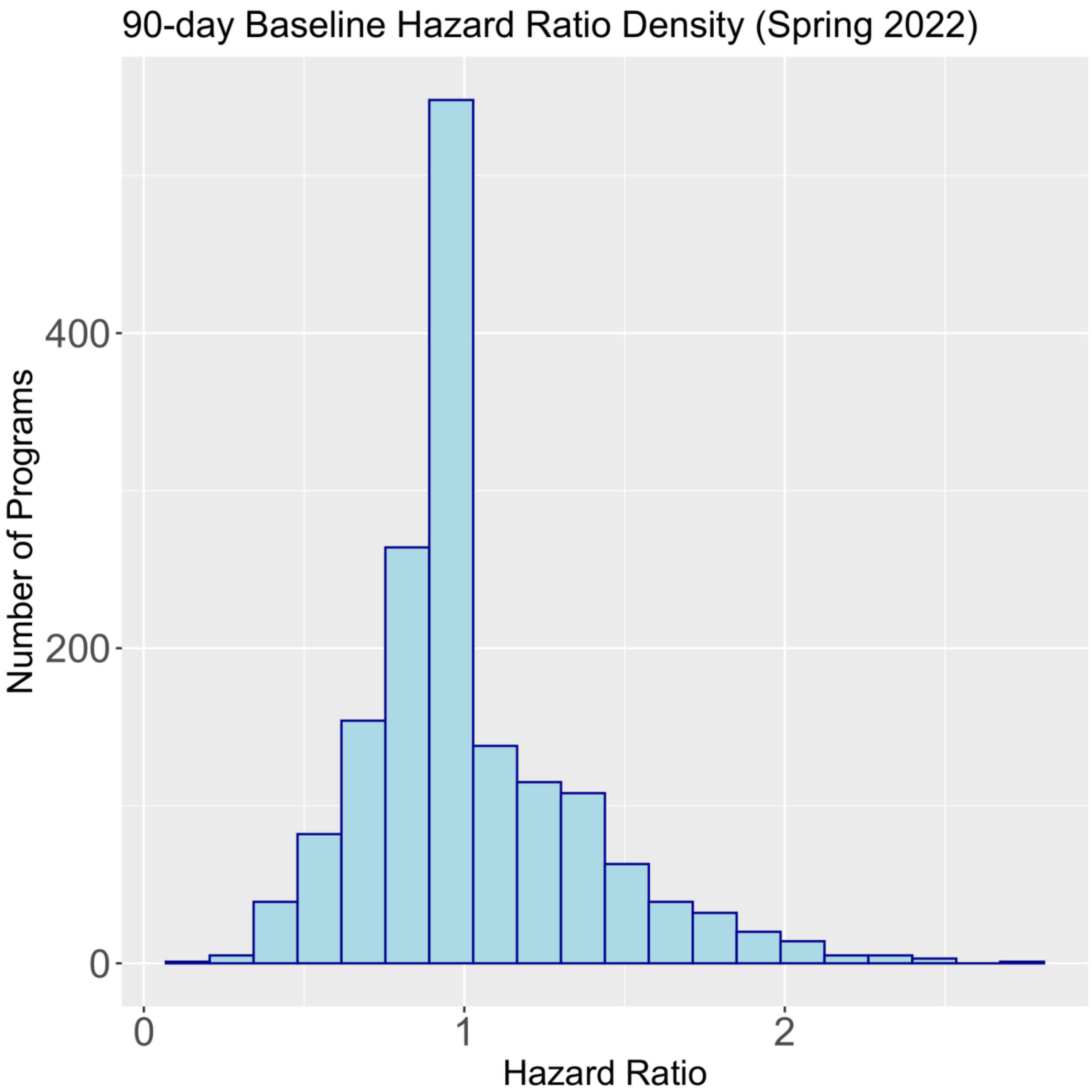

Supplemental Figure 2. 90-day Baseline Hazard Ratio Density, Spring 2022. Hazard ratios (HR) are provided for all programs (N=1,671). 1-year (1Y) hazard ratios ranged from 0.204 to 2.807 with a mean of 1.018, median of 0.964, and standard deviation of 0.339.

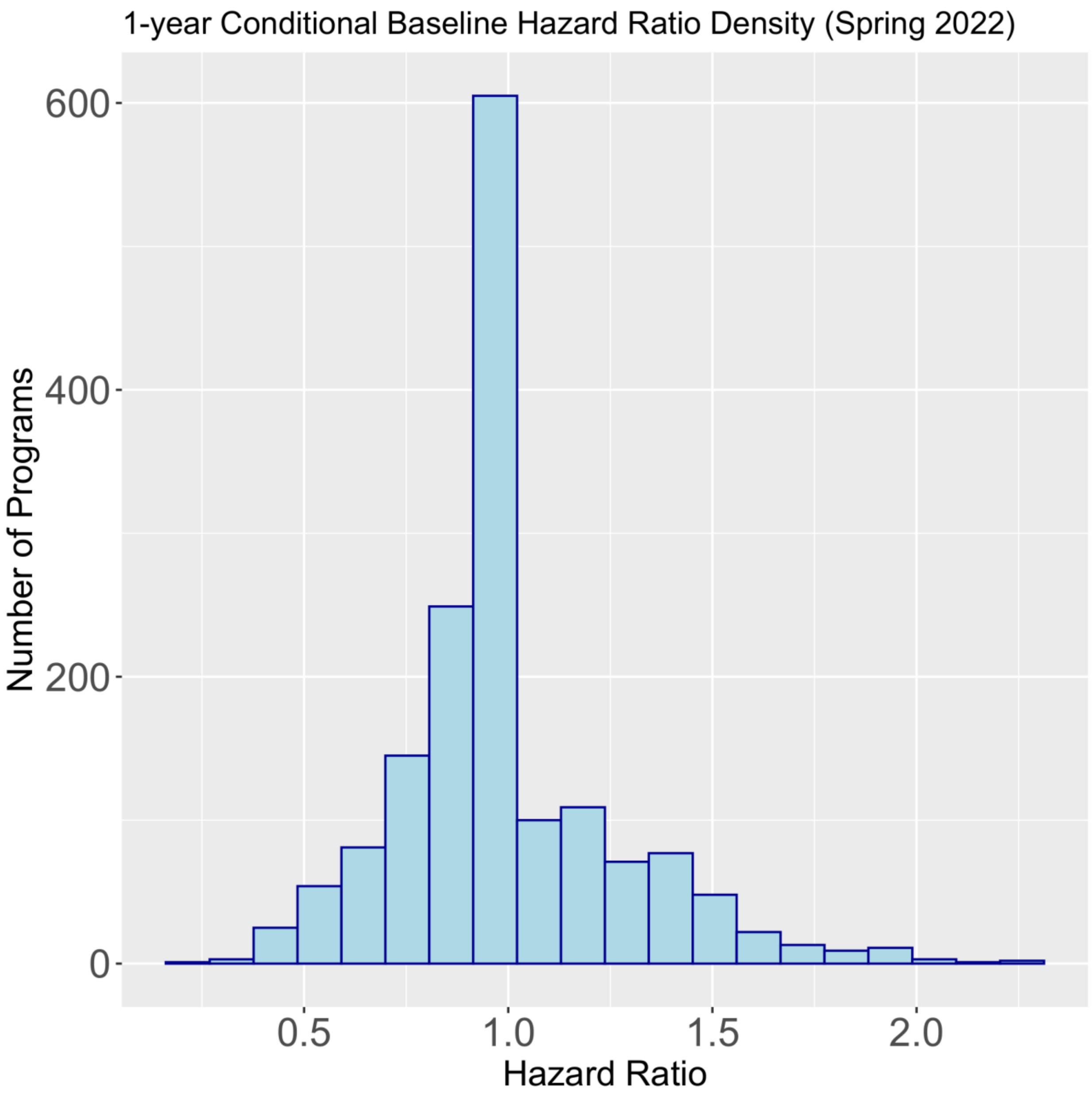

Supplemental Figure 3. 1-year Conditional Baseline Hazard Ratio Density, Spring 2022. Hazard ratios (HR) are provided for all programs (N=1,671). 1-year (1Y) hazard ratios ranged from 0.23 to 2.27 with a mean of 0.996, median of 0.973, and standard deviation of 0.269.

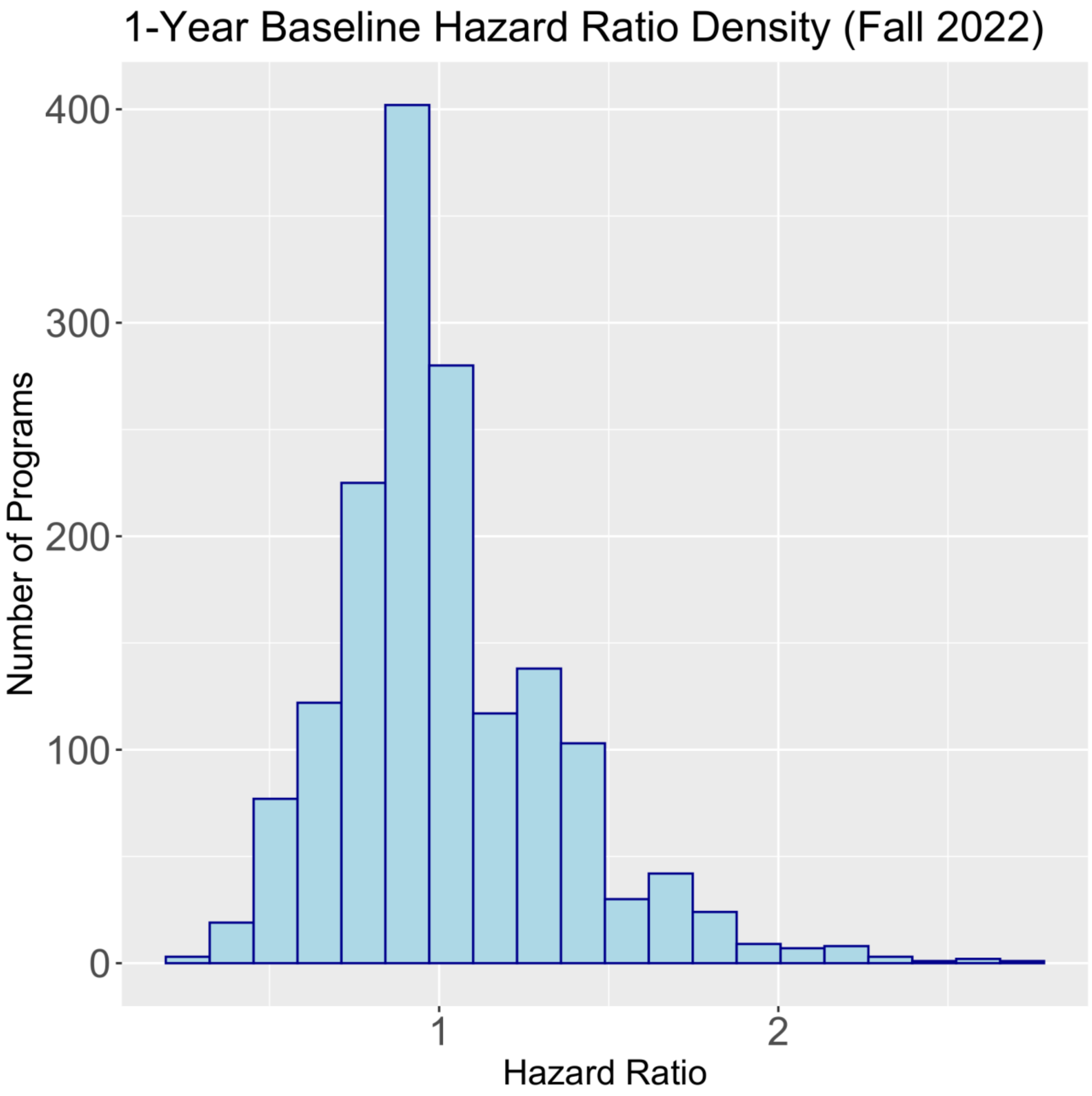

Supplemental Figure 4. 1-year Baseline Hazard Ratio Density, Fall 2022. Hazard ratios (HR) are provided for all programs (N=1,931). 1-year (1Y) hazard ratios ranged from 0.253 to 2.713 with a mean of 1.02, median of 0.96, and standard deviation of 0.331.

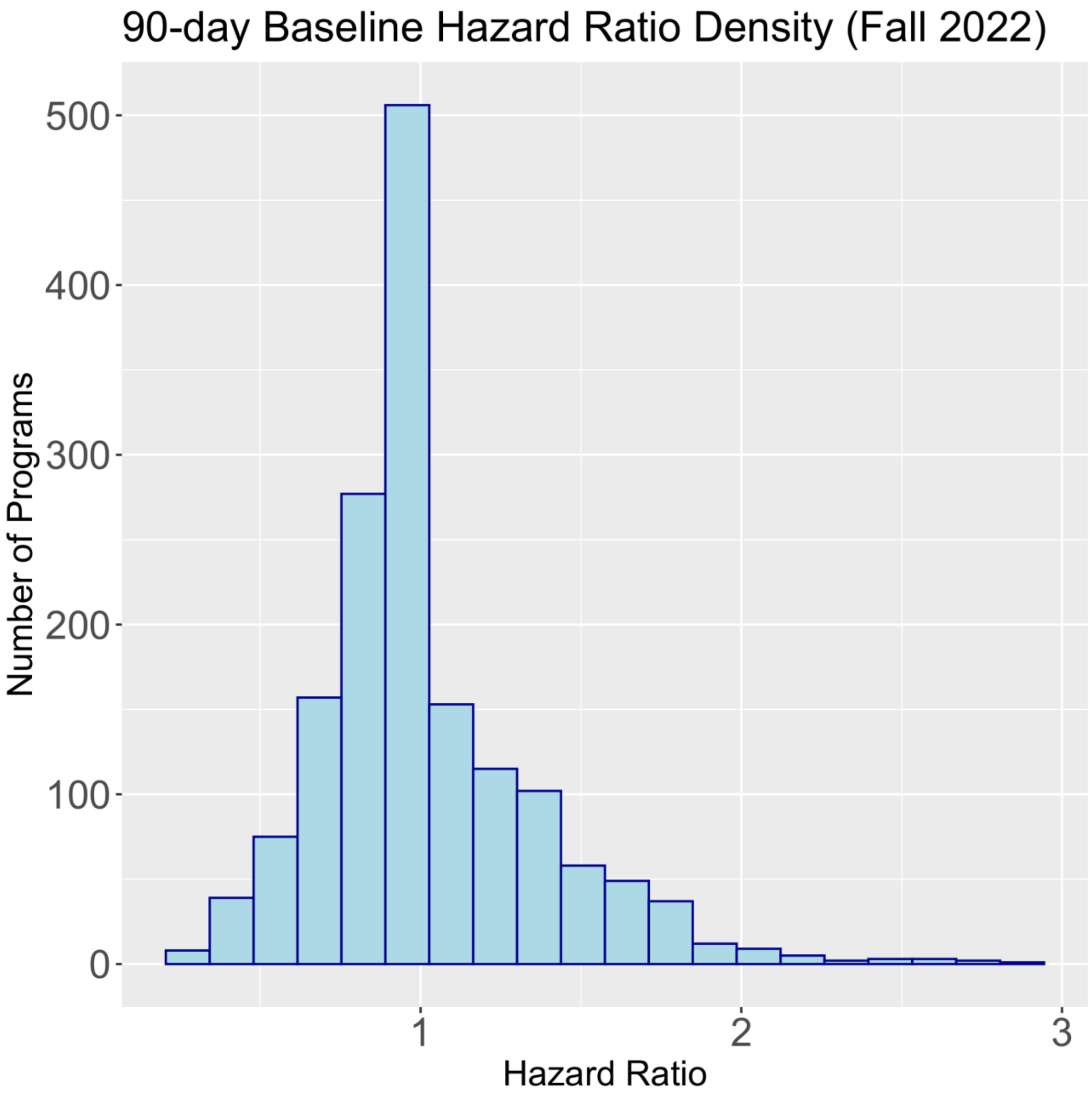

Supplemental Figure 5. 90-day Baseline Hazard Ratio Density, Fall 2022. Hazard ratios (HR) are provided for all programs (N=1,931). 1-year (1Y) hazard ratios ranged from 0.261 to 2.863 with a mean of 1.02, median of 0.961, and standard deviation of 0.344.

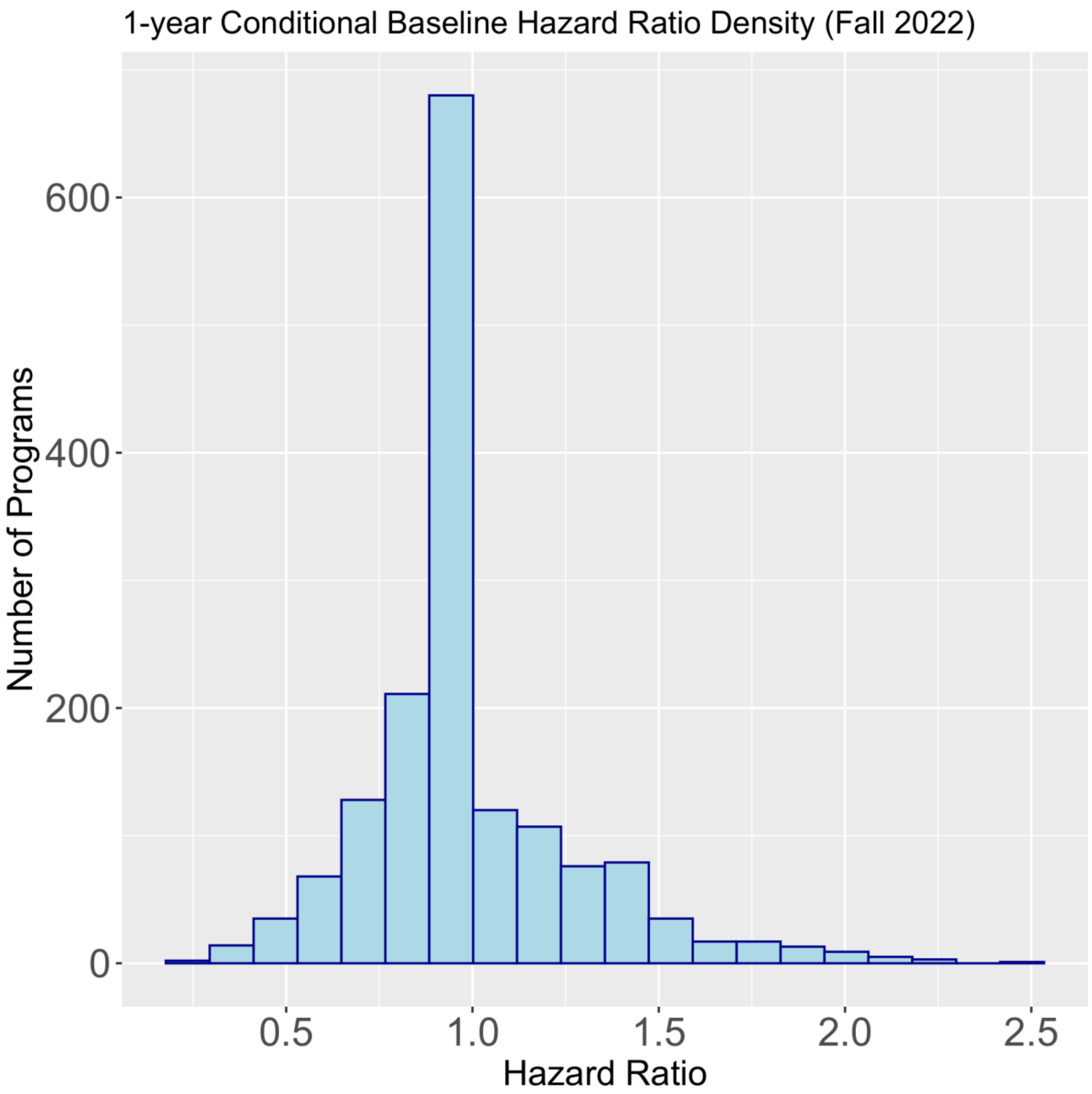

Supplemental Figure 6. 1-year Conditional Baseline Hazard Ratio Density, Fall 2022. Hazard ratios (HR) are provided for all programs (N=1,931). 1-year (1Y) hazard ratios ranged from 0.291 to 2.53 with a mean of 1, median of 0.974, and standard deviation of 0.281.

Baseline HR > 1

90D \ 1YC	TRUE	FALSE
TRUE	187	380
FALSE	386	648

Baseline HR > 1.25

90D \ 1YC	TRUE	FALSE
TRUE	87	232
FALSE	410	872

Baseline HR > 1.5

90D \ 1YC	TRUE	FALSE
TRUE	31	107
FALSE	322	1141

Baseline HR > 1.75

90D \ 1YC	TRUE	FALSE
TRUE	15	55
FALSE	295	1236

Baseline HR > 2

90D \ 1YC	TRUE	FALSE
TRUE	8	18
FALSE	224	1351

Supplemental Figure 7. Contingency Tables for 90-day versus 1-year Conditional Cohorts by Hazard Ratio, Spring 2022. Five contingency tables are provided for all programs (N=1,671) and organs with hazard ratios above five hazard ratio thresholds (1, 1.25, 1.5, 1.75, and 2). 90-day (90D) observations are on the y-axis and 1-year conditional (1YC) observations are on the x-axis for each table.

Baseline HR > 1

90D \ 1YC	TRUE	FALSE
TRUE	223	341
FALSE	339	667

Baseline HR > 1.25

90D \ 1YC	TRUE	FALSE
TRUE	116	203
FALSE	377	874

Baseline HR > 1.5

90D \ 1YC	TRUE	FALSE
TRUE	30	112
FALSE	329	1099

Baseline HR > 1.75

90D \ 1YC	TRUE	FALSE
TRUE	14	54
FALSE	304	1198

Baseline HR > 2

90D \ 1YC	TRUE	FALSE
TRUE	3	20
FALSE	251	1296

Supplemental Figure 8. Contingency Tables for 90-day versus 1-year Conditional Cohorts by Hazard Ratio, Fall 2022. Five contingency tables are provided for all programs (N=1,931) and organs with hazard ratios above five hazard ratio thresholds (1, 1.25, 1.5, 1.75, and 2). 90-day (90D) observations are on the y-axis and 1-year conditional (1YC) observations are on the x-axis for each table.

Supplemental Figure 9. Interdependence Between the New Post-transplant Evaluation Metrics. Correlation analysis for the Spring (top) and Fall 2022 (bottom) cohorts. The mean correlation coefficients between the 90D and 1YC90D cohorts for all organs (aggregate) ranged from -0.672 at HRs greater than 1.0 to -0.865 at HRs greater than 1.75 for the Spring 2022 cohort. Heart, kidney, liver, and lung correlation coefficients ranged from [-0.681 to -1], [-0.655 to –0.909], [-0.633 to -1], and [-0.455 to –0.679], respectively. For the Fall 2022 cohort, the aggregate correlation coefficients ranged from -0.605 to -0.927. Heart, kidney, liver, and lung HRs ranged from [-0.701 to -1], [-0.68 to –0.95], [-0.685 to -1], and [-0.514 to -1], respectively.

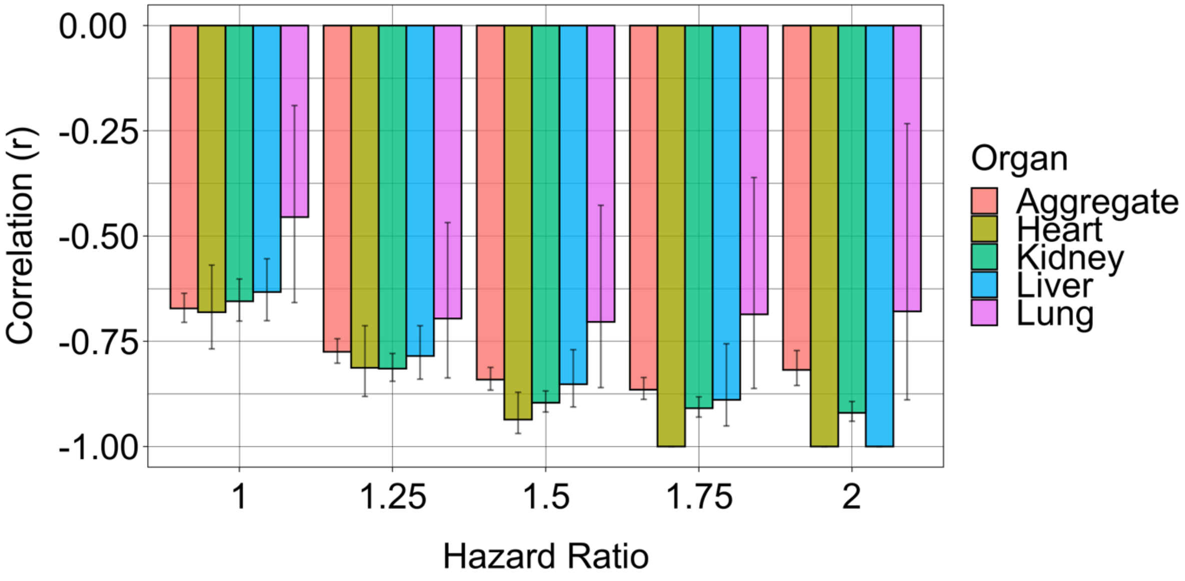

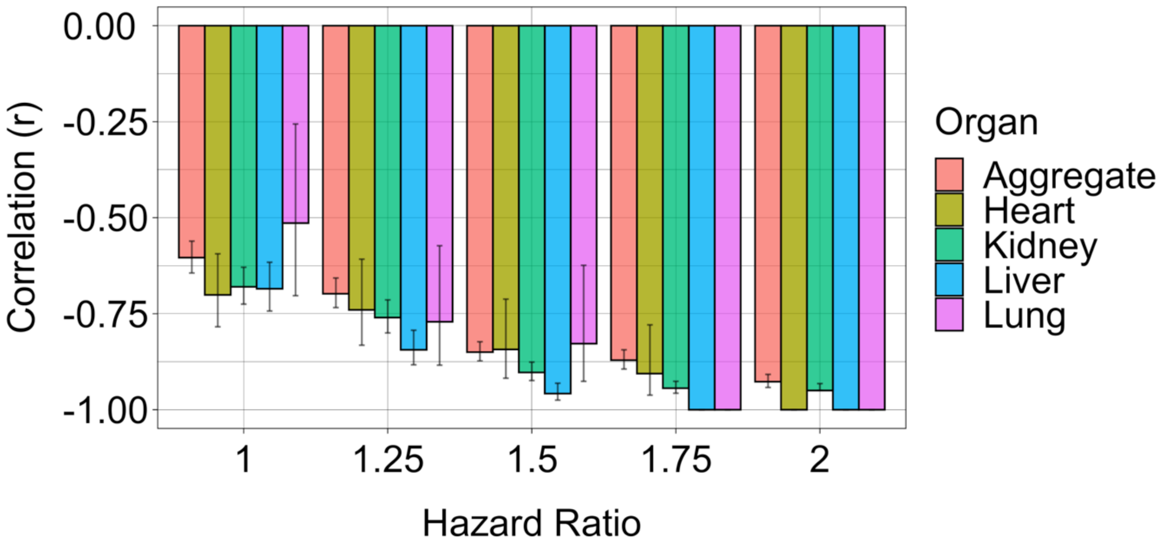

Supplement: Supplementary file 1 [file Datasheet1.pdf]
